# Renal Fibrosis: SIRT1 Still of Value

**DOI:** 10.3390/biomedicines12091942

**Published:** 2024-08-23

**Authors:** Huailiang Wu, Zhen Qiu, Liyan Wang, Wei Li

**Affiliations:** 1 Department of Anesthesiology, Renmin Hospital of Wuhan University, Wuhan 430060, China; whliang360@163.com (H.W.); qiuzhen124@126.com (Z.Q.); 2 Department of Obstetrics and Gynecology, Renmin Hospital of Wuhan University, Wuhan 430060, China; wlyan360@163.com

**Keywords:** renal fibrosis, SIRT1, lipid metabolism, oxidative stress, aging

## Abstract

Chronic kidney disease (CKD) is a major global health concern. Renal fibrosis, a prevalent outcome regardless of the initial cause, ultimately leads to end-stage renal disease. Glomerulosclerosis and renal interstitial fibrosis are the primary pathological features. Preventing and slowing renal fibrosis are considered effective strategies for delaying CKD progression. However, effective treatments are lacking. Sirtuin 1 (SIRT1), a nicotinamide adenine dinucleotide (NAD^+^)-dependent deacetylase belonging to class III histone deacetylases, is implicated in the physiological regulation and protection of the kidney and is susceptible to a diverse array of pathological influences, as demonstrated in previous studies. Interestingly, controversial conclusions have emerged as research has progressed. This review provides a comprehensive summary of the current understanding and advancements in the field; specifically, the biological roles and mechanisms of SIRT1 in regulating renal fibrosis progression. These include aspects such as lipid metabolism, epithelial-mesenchymal transition, oxidative stress, aging, inflammation, and autophagy. This manuscript explores the potential of SIRT1 as a therapeutic target for renal fibrosis and offers new perspectives on treatment approaches and prognostic assessments.

## 1. Introduction

### 1.1. Real Fibrosis

Renal fibrosis is the terminal pathological process of chronic kidney disease (CKD), characterized by excessive deposition of extracellular matrix (ECM) leading to scar formation. The mechanism of renal fibrosis involves a variety of factors, including injury to renal tubular epithelial cells, infiltration of inflammatory cells, and activation of myofibroblasts. In this process, TGF-β, Wnt, Notch, and Hedgehog signaling pathways are considered to be the major regulatory pathways in renal fibrosis, and they affect the onset and development of renal fibrosis through independent or interactive effects (Table 1). Its contributing factors including hypoxia, inflammation, aging, medications, metabolic disorders, and hypertension [1,2]. These factors signify the impaired healing response in kidney tissue following chronic injury. The development of renal fibrosis involves various types of renal cells. Throughout the progression of renal fibrosis, numerous cellular processes occur simultaneously and interrelate. These processes include the activation of mesangial cells and fibroblasts, epithelial-to-mesenchymal transition (EMT) of renal tubular cells, infiltration of mononuclear/macrophages and T cells, and apoptosis. In healthy adult kidneys, fibroblasts are situated within the interstitial spaces between capillaries and epithelial cells, creating a network across the renal parenchyma that contributes to the stabilization of the tissue architecture. Upon activation, fibroblasts proliferate and deposit a large amount of extracellular matrix (ECM), leading to the formation of fibrotic tissue. Excessive accumulation of ECM disrupts the normal structure and function of the kidneys, resulting in pathological alterations such as glomerulosclerosis, tubular damage, interstitial fibrosis, and vascular alterations [3]. Renal fibrosis is a complex pathological process, and the treatment of renal fibrosis is still in the exploratory stage. Several drugs such as RAS blockers and SGLT2 inhibitors have been used to slow the progression of CKD. Meanwhile, genetic and epigenetic changes, such as acetylation, DNA methylation, RNA interference, and chromatin remodeling, offer new opportunities for therapeutic strategies.

### 1.2. SIRT1

Histone deacetylase (HDAC) enzymes catalyze the deacetylation of both histone and non-histone proteins, significantly modulating both normal and disease-associated gene expression. Sirtuins, which constitute the class III HDACs, are instrumental in various biological processes, including DNA repair, apoptosis, regulation of the cell cycle, oxidative stress management, mitochondrial functionality, energy metabolism, lifespan extension, and aging [42]. The Sirtuin family comprises seven members, designated as SIRT1–7. The functions and positioning of SIRT1–7 are shown in Table 2 below. The first member, Silent Information Regulator 2 (Sir2), was originally discovered in yeast and influences a broad spectrum of cellular functions [43]. These include the regulation of telomere and ribosomal DNA (rDNA) silencing, modulation of intracellular signaling pathways associated with the cell cycle and aging, and metabolic control via the deacetylation of histones as well as numerous transcription factors and cofactors.

The SIRT1 protein, a highly conserved nicotinamide adenine dinucleotide (NAD^+^)-dependent deacetylase, belongs to the sirtuin family and is the closest mammalian ortholog to Sir2. It was the first deacetylase identified in mammals and has been the subject of extensive research. SIRT1, predominantly localized in the nucleus, can translocate to the cytoplasm under certain conditions, including ischemic stress and during embryonic development. The human SIRT1 gene is located on chromosome 10q22.1 and consists of nine exons and eight introns, encoding a protein of 747 amino acids. In contrast, mouse SIRT1 encodes a protein containing 737 amino acid residues. The SIRT1 protein is composed of an N-terminal domain, a catalytic domain, and a C-terminal domain. SIRT1 has a three-dimensional structure comprising a highly conserved Rossmann fold main domain and a secondary domain that includes a zinc-binding module and a helical module. The acetylated residue of the target molecule interacts with NAD^+^ within the cleft formed between the two domains, initiating a catalytic reaction. SIRT1 relies on NAD^+^ for its catalytic activity. Through the hydrolysis of NAD^+^ and the concurrent transfer of the acetyl group from the acetylated lysine residue of target proteins to the 2′-OH position of adenosine diphosphate (ADP)-ribose, SIRT1 catalyzes the deacetylation process, resulting in the formation of nicotinamide and 2′-*O*-acetyl-ADP-ribose. The activity of SIRT1 is modulated by several factors including the NAD^+^/NADH ratio, interactions with binding partners, and post-translational modifications [44].

Sirtuins play pivotal roles in various biological processes, including cell proliferation, mitochondrial energy balance, and antioxidant function. For example, SIRT7 alleviates renal ferritin deposition, lipid peroxidation, and pEMT under hypertensive conditions through the enhancement of the KLF15/ nuclear factor erythroid 2-related factor 2 (Nrf2) signaling pathway, which ameliorates renal fibrosis, injury, and dysfunction [45]. SIRT1 exerts a renoprotective effect in DKD in part through the deacetylation of transcription factors involved in disease pathogenesis (e.g., p53, FOXO, RelA/p65NF-κB, STAT3, and PGC1α/PPARγ), and SIRT1 agonists may be a novel therapy to halt the progression of DKD [46]. Multiple nephroprotective effects of SIRT1 make it equally attractive in the treatment of CKD [47].

In summary, most sirtuin family members exhibit regulatory effects on proteins associated with renal fibrosis. SIRT1, which has been extensively studied, regulates transcriptional activity by affecting the acetylation of histone and non-histone proteins. It can also modulate the biological activity of co-transcription factors or directly induce the deacetylation of signaling protein molecules. SIRT1 is a potential therapeutic target for renal fibrosis and is involved in aging, energy homeostasis, autophagy, mitochondrial biogenesis, and apoptosis [48]. As the ultimate destination of renal injury of various causes, renal fibrosis currently lacks effective prevention and treatment methods. With the understanding of the pathogenic mechanisms of renal fibrosis, the role of SIRT1 is also being updated, so we chose to conduct a review of the latest role of SIRT1 in renal fibrosis

### 1.3. The Impact of SIRT1 on Renal Fibrosis

**Figure 1:**
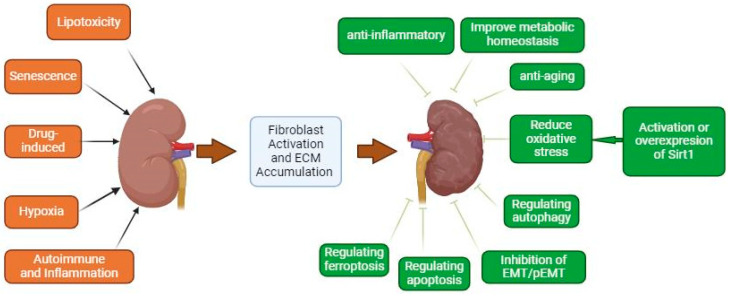
Risk factors for renal fibrosis and the mechanism of SIRT1 in renal fibrosis. Risk factors include drug-induced, autoimmune/inflammation, lipotoxicity, hypoxia, and senescence. SIRT1 mainly affects renal fibrosis in metabolic reprogramming, oxidative stress, inflammatory response, autophagy, aging, apoptosis, and EMT/pEMT.

Previous studies demonstrated that SIRT1 plays a role in the physiological regulation of the kidney, offering protection against various pathological factors (Figure 1). In mice subjected to unilateral ureteral obstruction (UUO), deletion of SIRT1 in renal interstitial cells exacerbates renal injury and fibrosis. SIRT1 exerts a protective effect on renal injury and fibrosis, potentially through the suppression of HIF-2α [49]. Additionally, SIRT1 downregulates type 1 angiotensin receptors and nuclear factor-kappa B (NF-κB), contributing to renal protection in UUO. Calcium oxalate (CaOx) stones, a prevalent form of kidney stones, are linked to renal tubular damage, interstitial fibrosis, and the progression of CKD. The induction of ferroptosis through SIRT1-mediated deacetylation of p53 may be a viable target for preventing renal fibrosis in patients with nephritis [50]. Although numerous studies have demonstrated the protective role of SIRT1 in acute renal injury and fibrosis, there is ongoing controversy regarding this matter. For example, studies have shown that the inhibition of SIRT1 and SIRT2 with Sirtinol in the UUO model alleviated renal fibrosis. Additionally, the specific inhibition of SIRT1 via EX527 (6-chloro-2,3,4,9-tetrahydro-1*H*-carbazole-1-carboxamide) or SIRT1 siRNA silencing effectively attenuated the activation of renal interstitial fibroblasts [51]. SIRT1 plays a crucial role in various biological processes, including metabolic homeostasis, oxidative stress, inflammatory responses, autophagy, aging, apoptosis, and EMT. This review discusses the role of SIRT1 in renal fibrosis by examining its involvement in the various biological processes associated with its development.

## 2. Potential Mechanism of SIRT1-Mediated Regulation of Renal Fibrosis

### 2.1. Metabolic Reprogramming

After acute kidney injury (AKI), substantial mitochondrial impairment and insufficient oxygen/nutrient delivery result in diminished glycolysis and mitochondrial energy metabolism. Concurrently, perturbations occurred in the pentose phosphate pathway (PPP), amino acid metabolism, and ketone body availability. During the ensuing renal repair phase, there is a metabolic transition towards glycolysis, a reduction in fatty acid β-oxidation, and persisting disturbances in amino acid metabolism. The critical features of AKI that lead to the transition to CKD involve myofibroblast proliferation and secretion. Myofibroblasts originate from various cell types including pericytes. Chen et al. [52] elucidated the metabolic reprogramming of pericytes during the pericyte-to-myofibroblast transition (PMT) and found that fatty acid oxidation was downregulated, whereas glycolysis was upregulated in pericytes during PMT. Inhibiting this transition can alleviate renal fibrosis. Therefore, multiple metabolic pathways undergo changes following kidney injury. Metabolic reprogramming is a pivotal characteristic of the renal repair process and profoundly affects renal cell injury, regeneration, and advancement of renal fibrosis [53]. The kidney’s energy metabolism encompasses various pathways that actively produce adenosine triphosphate (ATP) and other vital metabolites necessary for renal function. Renal cells from distinct segments of the nephron utilize specific metabolic pathways based on their unique functions and energy demands. The primary energy sources in healthy kidneys are glucose, fatty acids, amino acids, and ketones.

The substantial energy requirements of the proximal and distal tubules in the kidney are attributable to their critical functions in substance transport and reabsorption. In highly metabolic cells, fatty acid oxidation (FAO) is the preferred energy source because it generates more ATP per molecule than glucose oxidation. The transport of fatty acids into the mitochondria, a critical step in their metabolism, is facilitated by carnitine palmitoyltransferase 1 (CPT1), which catalyzes the conjugation of fatty acids with carnitine. CPT1 is the rate-limiting enzyme in FAO. Key transcriptional regulators, including peroxisome proliferator-activated receptors (PPARs) and PPAR-γ coactivator-1α (PPARGC1A), are pivotal in modulating the expression of proteins essential for fatty acid uptake and oxidation [54]. Under physiological conditions, fatty acid uptake, oxidation, and synthesis are meticulously balanced to prevent intracellular lipid accumulation.

In renal fibrosis, aberrant energy metabolism leads to metabolic reprogramming in kidney cells, including the activation of oxidative glycolysis and reduction in FAO, to adapt to metabolic stress. This metabolic reprogramming impairs renal cell function and accelerates fibrosis progression. Dysregulation of energy metabolism results in mitochondrial and peroxisomal dysfunction, which affects intracellular ATP synthesis and reactive oxygen species (ROS) production. Mitochondria are the primary energy sources for cells and are crucial for stable biosynthesis, whereas ROS regulate cellular oxidative stress, apoptosis, and gene expression. When kidney cells exhibit metabolic abnormalities and mitochondrial function is compromised, the increased intracellular ROS levels lead to oxidative damage and fibrosis [55].

SIRT1, the most evolutionarily conserved NAD^+^-dependent protein deacetylase in mammals, is a pivotal metabolic sensor in diverse metabolic tissues [56]. SIRT1 serves as a direct mediator between cellular metabolic status and modulation of chromatin architecture and gene expression, influencing diverse cellular functions, including energy metabolism and stress response mechanisms. Recent studies have indicated that SIRT1 is involved in the reprogramming of glucose and lipid metabolism [57]. Under conditions of low energy, it actively modulates PPARα and its coactivator PGC-1α, leading to the enhanced utilization of fatty acids. For instance cigarette smoking downregulates SIRT1, leading to dysregulation of lipid metabolism and activation of lung fibroblasts, thereby promoting the development of idiopathic pulmonary fibrosis, SIRT1 activator SRT1720 counteracted the effect of CS on NOX4, SOD2, PPARα and CPT1a in vivo [58]. Lipid accumulation in podocytes is a potential therapeutic target for diabetic nephropathy. Thus, baicalin may alleviate lipid metabolic abnormalities in the kidneys of db/db mice via the SIRT1/AMPK/HNF4A pathway, thereby exerting protective effects on the kidneys [59]. Furthermore, SIRT1 overexpression and SRT1720 treatment reduced renal lipid content, adipogenesis, oxidative stress, and inflammatory marker expression and, to some extent, alleviated renal interstitial fibrosis [60]. Previously, we observed a significant reduction in SIRT1 expression in mice with ischemia/reperfusion injury (IRI). SIRT1 overexpression enhances renal function and reduces lipid accumulation and renal fibrosis. Mechanistically, SIRT1 impedes the acetylation of histone H3K27 at the ACLY promoter, thereby suppressing FAO activity and promoting renal fibrosis. SP1 modulates FAO by directly influencing SIRT1 expression [61]. Song et al. [62].demonstrated CPT1A-mediated FAO via the SIRT1/STAT3/Twist1 pathway, thereby inhibiting EMT in renal TECSs. Furthermore, FAO suppression precedes EMT, and EMT is an outcome of FAO inhibition. FAO is a pivotal modulator of cellular senescence and is a substantial contributor to renal fibrosis. The ablation of FAO triggers p53-mediated cellular senescence in human fibroblasts, whereas the augmentation of FAO activity mitigates replicative senescence. Inhibition of autophagy or overexpression of SIRT1 can alleviate senescence resulting from FAO inhibition [63].

**Figure 2:**
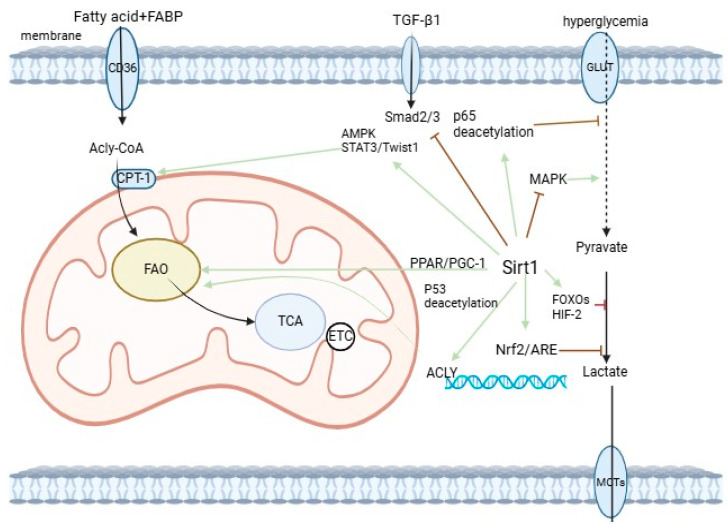
Role of SIRT1 in glycolipid metabolism. SIRT1-mediated deacetylation of some transcription factors (e.g., NF-κB, FOXOs, p53) may be involved in the protective effects of metabolic reprogramming. Overall, SIRT1 alleviates kidney injury, EMT, and aging by promoting fatty acid metabolism, inhibiting glycolysis, and mitigating lipotoxicity.

Hyperglycemia-mediated oxidative stress, inflammation, apoptosis, and EMT play crucial roles in the pathogenesis of renal fibrosis. Studies have shown that SIRT1 overexpression in capsular and renal tubular cells in animal models of diabetic kidney disease (DKD) can alleviate proteinuria and renal injury. The renoprotective effects of SIRT1 in DKD are partly due to the deacetylation of transcription factors involved in disease pathogenesis, such as NF-κB, Smad3, FOXO, and p53. Research has demonstrated that SIRT1 can activate the Nrf2/ARE signaling pathway, thereby alleviating oxidative stress in diabetic nephropathy and preventing the progression of renal fibrosis [64]. Moreover, upregulating SIRT1 expression can enhance SIRT1-dependent deacetylation of HIF-1α, reduce HIF-1α activity, and ultimately improve renal EMT and diabetic interstitial fibrosis in the kidney [65]. The kidneys are the most susceptible to damage under diabetic conditions. SIRT1, functioning as an energy sensor, plays a regulatory role in renal function, primarily by promoting the deacetylation of multiple lysine sites on PGC-1α to facilitate the restoration of cellular energy homeostasis. Increasing evidence suggests that hyperglycemia leads to reduced SIRT1 activity, resulting in increased acetylation of PGC-1α and dysfunction in energy metabolism. These findings highlight the pivotal role of SIRT1 in glucose and lipid metabolism (Figure 2).

In summary, excessive lipid accumulation leads to increased cellular lipotoxicity and ROS levels, triggering oxidative stress, mitochondrial dysfunction, and endoplasmic reticulum stress, which contribute to renal cell senescence and interstitial fibrosis. Balancing fatty acid uptake, lipid synthesis, and degradation and improving lipid metabolism disorders can help delay renal aging and alleviate interstitial fibrosis. SIRT1, which plays a crucial role in metabolism, particularly glycolipid metabolism in the kidney, is a promising target for further studies.

### 2.2. Inflammatory Responses

Renal inflammation serves initially as a defensive response to kidney damage, aiming to eliminate harmful agents and facilitate tissue repair. However, persistent inflammation and activation of intrinsic renal cells lead to the secretion of profibrotic cytokines and growth factors. These factors recruit and activate myofibroblasts, which promote progressive glomerular and interstitial fibrosis, ultimately leading to end-stage renal disease. CKD is characterized by persistent inflammation, fibrosis, and renal function decline. Extensive evidence supports the pivotal role of inflammation in initiating and advancing renal fibrosis 

In AKI, transient renal ischemia can induce responses similar to those seen in CKD, including elevated cytokine release, inflammatory cell infiltration, EMT, and fibroblast activation. The initial tubular damage is subsequently repaired via tubular regeneration and matrix remodeling. However, unresolved initial fibrosis can reoccur later due to factors such as hypertension, diabetes, or immune system activation. Fibrotic signaling not only triggers fibroblast activation but also initiates tubular EMT, a critical event that contributes to renal fibrosis and the progression to CKD [66].

Increasing evidence has indicated that SIRT1 expression is diminished during acute inflammatory responses and in diseases associated with inflammation, both in vivo and in vitro. Specific drugs and upstream molecules exert significant anti-inflammatory effects by enhancing SIRT1 levels [67,68] Overexpression of SIRT1 or increased SIRT1 activity significantly inhibits cytokine production and reduces inflammation in various animal models. Chronic inflammation can lead to maladaptive renal repair and fibrosis. Following repeated low-dose cisplatin (RLDC) injury, SIRT1 is downregulated, which results in the acetylation of p65, thereby activating NF-κB and perpetuating inflammatory responses. Activation of SIRT1 via agonists significantly reduces the acetylation of p65 (a key component of NF-κB), thereby alleviating inflammatory and fibrotic responses. Conversely, SIRT1 knockout exacerbated these cellular alterations. Indeed, activation of the NF-κB signaling pathway is pivotal in mediating the inflammatory response and represents one of the most thoroughly investigated pathways in inflammation research. Deacetylation of SIRT1 at lysine 310 reduces the acetylation of p65, which in turn diminishes the expression of inflammatory cytokines mediated by NF-κB, exerting an anti-inflammatory effect. Furthermore, SIRT1 can indirectly inhibit NF-κB signaling by regulating the expression of key proteins like AMPK and PPARγ [69,70].

HMGB1, a danger-associated molecular pattern (DAMP), exerts potent proinflammatory effects when released from necrotic cells. It has been reported that SIRT1 can suppress the activity of HMGB1 and that SIRT1 physically interacts with various lysine residues located at the HMGB1 nuclear localization signal (NLS) through its N-terminal lysine residues, resulting in the deacetylation of HMGB1. Consequently, HMGB1 is retained in the nucleus and its translocation to the cytoplasm is diminished [71]. HMGB1 inhibition alleviates renal injury and chronic renal fibrosis induced by I/R.

HIF-1α functions as a crucial transcription factor in the context of oxidative stress and pro-inflammatory reactions. SIRT1 interacts directly with HIF-1α, facilitating the deacetylation of HIF-1α at Lys374. This interaction results in the inactivation of HIF-1α by impeding the recruitment of the P300 acetyltransferase under hypoxic conditions. Research indicates involvement of SIRT1/HIF-1α in renal fibrosis [72].

In general, SIRT1-mediated histone deacetylation within the promoter regions of target genes directly impedes their transcription, thereby serving as a critical mechanism by which SIRT1 downregulates the expression of inflammatory cytokines. SIRT1 modulates inflammation through various signaling pathways, including NF-κB, HIF-1α, HMGB1, PPAR, and AMPK, which play crucial roles in the pathogenesis of renal fibrosis.

### 2.3. Oxidative Stress

Oxidative stress arises from an imbalance characterized by an excessive generation of free radicals due to inflammation and mitochondrial dysfunction, and a decline in antioxidant defense capabilities. It is characterized by the detrimental effects of excessive free radicals within the body, which can directly or indirectly damage DNA, proteins, and lipids.

Studies have shown that oxidative stress plays a critical role in the pathogenesis of renal fibrosis beyond its association with inflammation [73,74]. Oxidative stress and inflammation are intricately linked, creating a self-perpetuating loop, in which oxidative stress initiates inflammation via multiple molecular pathways. Extensive research has indicated that the overactive NF-κB signaling pathway is pivotal in the pathogenesis of renal fibrosis [75]. An overactive NF-κB pathway results in the activation and mobilization of immune cells. Conversely, inflammation, through the activation of leukocytes and resident cells, produces reactive oxygen and nitrogen species, which in turn exacerbate oxidative stress. These processes contribute to organ damage by promoting apoptosis, necrosis, and fibrosis. In most cases, NF-κB is maintained in an inactive state within the cytoplasm, where it forms a complex with its inhibitor proteins. Upon cellular stimulation, the inhibitor proteins dissociate from the complex, allowing NF-κB to become activated and translocate to the nucleus, where they regulate the transcriptional activation of target genes. SIRT1 inhibits the gene transcription of NF-κB by directly deacetylating lysine 310 on the P65/RelA subunit. SIRT1 activator SRT1720 restores the expression and activity of SIRT1 in diabetic mice and renal TECs subjected to high glucose-induced fibrosis models. Furthermore, SRT1720 inhibits NF-κB activation, thereby attenuating renal inflammation and fibrosis associated with diabetes. Yang et al. [76] also discovered that SIRT1 activation could ameliorate UUO-induced inflammation and renal interstitial fibrosis by reducing the expression of NF-κB, thereby minimizing the generation of reactive oxygen species.

Activation of the Kelch-like ECH-associated protein 1 (Keap1)/Nrf2/antioxidant response element (ARE) signaling pathway is pivotal in the cellular response to oxidative stress and effectively impedes the progression of renal fibrosis. Studies have reported that elevated SIRT1 levels in both in vivo and in vitro models enhance the activation of the Keap1/Nrf2/ARE pathway, facilitate the nuclear translocation of Nrf2, augment ARE- binding and transcriptional activity, and upregulate the expression of target genes to treat diabetic nephrogenic fibrosis [77]. Further investigation revealed that SIRT1 significantly enhanced the activity of the Keap1/Nrf2/ARE pathway to alleviate advanced glycation end-product-induced renal fibrosis. Additionally, Nrf2 upregulates SIRT1 protein expression and deacetylase activity, demonstrating the establishment of a positive feedback mechanism involving SIRT1 and the Keap1/Nrf2/ARE pathway, which is essential for suppressing diabetic nephrogenic fibrosis.

ROS play a pivotal role in fibrosis pathogenesis. The renal tubulointerstitium, which is characterized by high oxygen consumption for active solute transport and reabsorption, is particularly vulnerable to oxidative stress. Elevated ROS levels contribute to the development of renal fibrosis and have been implicated in a wide spectrum of kidney disorders. ROS-induced oxidative stress mediates cellular responses in multiple cell types induced by TGF-β1. SRT1720, a SIRT1 activator, confers protection against renal tubulointerstitial fibrosis induced by UUO, partially through the suppression of the renal ROS/TGF-β1/CTGF signaling cascade [78].

### 2.4. Epithelial-to-Mesenchymal Transition

Epithelial cells downregulate epithelial features and adopt mesenchymal properties through EMT. During this process, epithelial cells undergo a loss of intercellular junctions and apicobasal polarity, reorganize their cytoskeletal structures, and modify the signaling pathways that govern cell morphology and reprogram gene expression. EMT plays a crucial role in developmental processes and is reactivated during wound healing, fibrosis, and cancer progression. In the pathogenesis of renal fibrosis, EMT in renal TECs is characterized by phenotypic changes, including loss of cell adhesion, adoption of junctional proteins such as E-cadherin, and expression of mesenchymal cell markers. EMT results in the loss of functional parenchyma by impairing the proliferative capacity and functional characteristics of the renal TECs [79]. Damaged renal TECs exhibit significant metabolic rearrangements such as profound inhibition of FAO, which greatly affects regeneration. Renal TECs undergoing EMT do not transdifferentiate into interstitial fibroblasts; rather, they adhere to the renal tubular basement membrane and display a partial EMT phenotype. These partially EMT-transformed renal TECs stimulate the activation of interstitial fibroblasts and the recruitment of immune factors via a paracrine signaling pathway. One contentious issue is the lack of evidence demonstrating that renal TECs can traverse the basement membrane to become interstitial fibroblasts in the kidney. Recent developments in understanding renal fibrosis have focused on the innovative concept of “partial epithelial-mesenchymal transition (pEMT)” to clarify the role of renal TECs in the progression of renal fibrosis [80]. Studies have shown that renal TECs, upon injury, gain mesenchymal traits and the capacity to secrete various profibrotic factors and cytokines while maintaining their attachment to the basement membrane. This phenomenon has led to the introduction of pEMT, which elucidates the role of renal epithelial cells in the development of renal fibrosis. Despite controversies surrounding the role of EMT in renal fibrogenesis, as observed in various lineage-tracing studies, these discrepancies may arise from different experimental conditions, such as distinct disease models, mouse strains, and the types of genetic alterations employed.

Sirtuins are associated with the regulation of hypoxic responses, hypoxia-inducible factor (HIF)-1α and HIF-2α activation, and the induction of epithelial-mesenchymal transition (EMT). Interestingly, various sirtuins display differential effects on hypoxia and EMT, functioning as either stimulators or suppressors depending on specific tissue and cell contexts [81]. Studies have indicated that Sirtuin 1 (SIRT1) is an attractive therapeutic target for reversing EMT and tumor metastasis [82]. During development, wound healing, and diseases such as fibrosis, Transforming growth factor-β (TGF-β) plays a crucial role as an inducer of EMT [83]. SIRT1 inhibits the TGF-β/SMAD pathway by deacetylating SMAD2/3/4/7 [84]. Yang et al. [85] found that trimetazidine inhibits TGFβ1-induced EMT and renal fibrosis in a diabetic rat model via SIRT1-dependent deacetylation of smd4. YY1 (Yin Yang 1), a ubiquitous multifunctional zinc finger transcription factor with DNA/RNA-binding capabilities, is subject to regulation through acetylation and deacetylation modifications. Studies have shown that YY1 plays a crucial role in the regulation of renal fibrosis. SIRT1 inhibits EMT in diabetic nephropathy renal tubular cells via YY1 deacetylation [86]. SIRT1 plays a critical role in autophagy. SIRT1 mediates EMT regulation by modulating autophagy levels, and salvianolic acid B mitigates EMT in rats with renal fibrosis by activating SIRT1-induced autophagy [87,88]

EndoMT is a distinct form of EMT that describes the phenotypic transformation of endothelial cells into fully mesenchymal cells, potentially contributing to the myofibroblast pool in fibrosis [89]. TGF-β signaling and the activation of its downstream canonical/SMAD-mediated and non-canonical pathways have been identified as primary drivers of this process. SIRT1 may play a crucial role in inhibiting the TGF-β/SMAD pathway. Resveratrol (RSV)-activated SIRT1 modulates EndoMT through the TGF-β/SMAD2/3 pathway, thereby alleviating isoproterenol-induced cardiac fibrosis [90]. Wang et al. [91] found that calcium dobesilate has a potential role in renal interstitial fibrosis in obstructive nephropathy via inhibition of the EndoMT through activation of the SIRT1/p53 pathway. However, despite evidence of EndoMT in human renal biopsies and animal models of renal fibrosis, the degree to which EndoMT genuinely contributes to the myofibroblast population remains controversial [92].

### 2.5. Aging

Aging is a biological phenomenon characterized by a progressive decline in an organism’s structure and function with advancing age. Identification of this element as a pivotal risk factor for a spectrum of human diseases, including fibrotic disorders, has become increasingly evident [93,94]. Cellular senescence is a condition of irreversible cell cycle arrest marked by the enduring loss of proliferative potential while maintaining cellular viability and metabolic activity. This process is accompanied by gradual deterioration in autophagy, regulation of energy metabolism, stress tolerance, and overall metabolic health. Senescent cells cease to divide yet remain viable, secreting a range of factors collectively known as the senescence-associated secretory phenotype (SASP). SASP encompasses various elements including cytokines, chemokines, growth factors, and proteases. Initially, the SASP aids in cell cycle arrest and recruits immune cells to eliminate damaged or oncogene-expressing cells, thereby facilitating tissue repair. However, when repair mechanisms fail, the accumulation of senescent cells leads to the sustained secretion of SASP factors, creating a positive feedback loop. Persistent SASP secretion induces a chronic inflammatory state that activates myofibroblasts and promotes aberrant extracellular matrix deposition. These processes culminate in varying degrees of renal fibrosis, which ultimately results in renal dysfunction and age-related diseases [95,96]. Renal TEC senescence plays a pivotal role in the progression of AKI, impeding renal regeneration and repair mechanisms, and facilitating the progression of AKI to CKD via a SASP [97]. Furthermore, the similarities between SASP and CKD-associated secretory phenotype (CASP) are remarkable. Both phenotypes arise from irreversible cell cycle arrest and the induction of low-dose SASP, which, under the influence of genomic damage and external environmental cues, triggers elevated oxidative stress and a persistent inflammatory state. Similar to SASP, CASP components stimulate tissue repair via multiple pathways. However, when tissue repair is unsuccessful, the buildup of SASP or CASP elements intensifies the DNA damage response (DDR), thereby promoting the heightened release of SASP factors from senescent cells. This process accelerates cellular senescence, culminating in a range of age-related changes. Alterations in the microenvironmental components of renal tissue associated with SASP play a significant role in the development of renal fibrosis. The production of SASP involves intricate molecular and signaling pathways, including NF-κB, C/EBP-β, ATM-TRAF6-TAK1, among others. Activation of these pathways leads to the generation and amplification of the SASP, thereby exacerbating inflammatory and fibrotic processes.

During cellular senescence, the expression of SIRT1 decreases, whereas the expression of SASP components increases. Subsequent investigations have shown that the acetylation levels of histone H3 at lysine 9 (H3K9) and histone H4 at lysine 16 (H4K16) within the promoter regions of interleukin (IL)-8 and IL-6 progressively increase during cellular senescence. These observations implied that SIRT1 inhibited the expression of SASP factors by mediating histone deacetylation in these promoter regions.

SIRT1 also catalyzes the deacetylation of non-histone substrates such as p53, FOXOs, PGC1-α, PPAR-γ, and NF-κB, altering their transcriptional and enzymatic activities as well as protein levels, thereby extensively participating in the regulation of cellular senescence and organismal lifespan. Sung et al. [98] confirmed that SRT1720, a selective SIRT1 activator, attenuates cellular senescence and inflammatory cytokine secretion in human dermal fibroblasts through the enhancement of NF-κB deacetylation and autophagy activation. Furthermore, SIRT1-mediated deacetylation of HIF-1α exhibits potential protective effects against tubulointerstitial damage in aging kidneys [99]. Research has shown that SIRT1 activation and p53 deacetylation have been identified as potential strategies for alleviating premature renal aging and fibrosis [100]. Aging is characterized by mitochondrial dysfunction and oxidative stress. Activation of AMP-activated protein kinase (AMPK) and SIRT1 allows their target molecules to undergo simultaneous deacetylation and phosphorylation, thereby reducing the sensitivity of the kidney to age-related changes. Targeting SIRT1 can prevent or reduce age-related pathological changes in the renal tissue, including renal fibrosis. For another aspect, caloric restriction (CR) confers multiple health benefits such as lifespan extension, with one of its mechanisms as the mitigation of mitochondrial dysfunction across diverse pathological states. SIRT1 levels are notably diminished in aged kidneys but are elevated following prolonged CR. Extended CR promotes the deacetylation of FOXO3 through the upregulation of SIRT1 in aged mammalian tissues, thereby augmenting autophagy and ameliorating tissue damage and fibrosis associated with aging and hypoxia [101].

### 2.6. Autophagy

Autophagy is the primary degradation system within cells in which cytoplasmic materials are transported to lysosomes for degradation. However, autophagy serves not only as a mechanism for the removal of cellular components but also as an intricate recycling process that provides essential building blocks and energy for cellular restructuring and maintenance of homeostasis. Reduced autophagy is a hallmark of aging [102]. Autophagy mitigates renal fibrosis through the suppression of cellular inflammation, immune regulation, oxidative stress control, reduction of ECM deposition, and inhibition of the profibrotic factor TGF-β1. Increased expression of SIRT1 stimulates the basal rate of autophagy. SIRT1 forms molecular complexes with pivotal components of the autophagy machinery, such as Atg5, Atg7, and LC3-associated Atg8, and subsequently deacetylates these essential autophagy factors to induce autophagy. Furthermore, SIRT1 deacetylates the transcription factor FOXO3a, resulting in the upregulation of the pro-autophagic protein Bnip3 (Bcl-2/adenovirus E1B 19-kDa-interacting protein 3). The deacetylase activity of SIRT1 serves as a critical modulator of autophagy in vivo. Additionally, SIRT1 may facilitate the phosphorylation of LKB1, leading to the activation of AMPK, thereby initiating autophagy [103]. Reduced SIRT1 expression, a key enhancer of autophagy, in diabetic kidneys may contribute to the dysregulation of autophagy. Moreover, AMPK phosphorylation, which is positively correlated with SIRT1 activity and autophagy, is reduced in diabetic kidneys. Dietary restriction (DR) enhances autophagy, resulting in the normalization of mitochondrial morphology and reduction of p62/Sqstm1 accumulation, along with the upregulation of SIRT1 expression and activation of the AMPK pathway. Therefore, SIRT1 may play a role in DR by restoring SIRT1 protein expression in the kidneys, thereby improving autophagy dysregulation, counteracting inflammation, and alleviating renal damage in patients with diabetes. Increasing evidence suggests a direct link between autophagy and EMT. Overexpression of SIRT1 in renal cells has been reported to attenuate EMT and fibrosis by promoting autophagy. He et al. [87] demonstrated that salvianolic acid B mitigates EMT in renal fibrosis by inducing SIRT1-dependent autophagy studies analogous to ours, which have demonstrated the efficacy of targeting the SIRT1-NF-κB pathway and autophagy activation in modulating EMT in podocytes, thereby ameliorating renal fibrosis. Reports have also indicated autophagy activation in animal models of renal fibrosis. Similar studies have demonstrated the detrimental role of autophagy in diabetic nephropathy and adriamycin (ADR)-induced renal fibrosis [104]. However, the exact role of autophagy, whether it is negative or positive, is complex. NAD^+^ homeostasis is closely linked to autophagy, a conserved pathway for the clearance of damaged organelles. Autophagy plays a crucial role in renal homeostasis and survival during IRI. Therefore, induction of autophagy may enhance the therapeutic efficacy of potential treatments. However, this balance is challenging because autophagy can promote cell survival and induce cell death, potentially exacerbating IRI in the kidneys. Thus, the protective or detrimental nature of autophagy depends on the duration of the injury.

### 2.7. Apoptosis

Apoptosis plays an important role in tissue homeostasis. Morphological changes leading to the fragmentation of cells, organelles, and DNA, as well as the formation of apoptotic bodies, are key characteristics of apoptosis [105]. The primary mechanisms involve endogenous and exogenous pathways and endoplasmic reticulum stress (ERS). Toxic substances or DNA damage-induced disruption of cellular homeostasis or imbalance triggers the intrinsic pathway of apoptosis. The activation of cell surface death receptors (DR) or withdrawal of dependent receptor ligands triggers the extrinsic pathway of apoptosis. ERS can mediate apoptosis through four primary pathways: protein kinase R-like endoplasmic reticulum kinase (PERK), inositol-requiring kinase 1α (IRE1α), activating transcription factor 6 (ATF6), and Ca^2+^. Numerous studies have confirmed the involvement of apoptosis in renal injury pathophysiology [106,107]. Apoptosis is considered a crucial mechanism involved in fibrotic diseases, and various interventions targeting fibroblast and epithelial cell apoptosis are regarded as effective methods to delay fibrosis [108]. SIRT1 deacetylates multiple apoptosis-related proteins, including p53, SMAD7, FOXO3, and FOXO1, thereby protecting the renal cells from injury-induced apoptosis. p53, a pivotal tumor suppressor protein, functions as a crucial modulator of apoptosis triggered by diverse cellular stresses. Activation and stabilization of p53 are achieved via post-translational modification pathways, including ubiquitination, phosphorylation, and acetylation. SIRT1 inhibits p53 activity through deacetylation, and evidence suggests that p53 suppression reduces hyperglycemia-induced apoptosis in TECs, calcium oxalate, and cisplatin. In renal podocytes, advanced glycation end products (AGEs) downregulate SIRT1 expression, while SIRT1 overexpression counteracts AGE-mediated podocyte apoptosis by diminishing the acetylation of FOXO4. TGF-β has been demonstrated to be a crucial regulator of cell proliferation, differentiation, apoptosis, immune responses, and ECM production. SIRT1 modulates the TGF-β1 signaling pathway and attenuates apoptosis in mesangial cells exposed to TGF-β1. ER stress and UPR pathways significantly contribute to organ fibrosis by activating pro-apoptotic pathways, inducing EMT, and promoting inflammatory responses. According to previous reports, upregulation of SIRT1 reduces endoplasmic reticulum stress and renal fibrosis [109]. Interestingly, apoptosis appears to be a double-edged sword in renal fibrosis. Following physiological wound healing, myofibroblasts typically undergo programmed cell death and self-clearance. However, myofibroblast apoptosis is notably deficient under fibrotic conditions. Studies have shown that during fibrosis, myofibroblasts assume an anti-apoptotic and highly proliferative phenotype, resulting in the sustained activation of these cells and the continuation of the fibrotic disease process [110]. Targeting fibroblast apoptosis is a therapeutic approach for antifibrotic treatment [111]. Bulvik et al. [112] found that SIRT1 modulates persistent FLIP levels through miR-34a-mediated regulation and deacetylation of Ku70 in lung myofibroblasts, thereby enhancing resistance to apoptosis and contributing to lung fibrosis. Consequently, SIRT1 deficiency, especially in fibroblasts, diminishes the resistance to apoptosis and mitigates fibrosis.

### 2.8. Ferroptosis

**Figure 3:**
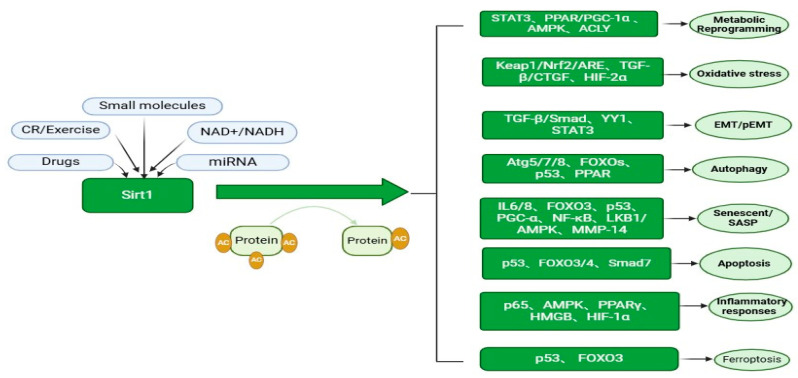
Potential mechanism of SIRT1-mediated regulation of renal fibrosis, and the factors that influence SIRT1 activity.

Iron plays essential physiological roles, including its involvement in the mitochondrial respiratory chain and hemoglobin synthesis. Excessive iron accumulation can trigger free radical production, leading to cellular organelle stress. Ferroptosis is a type of cell death that falls under the category of regulated cell death (RCD). Characterized by iron accumulation and lipid peroxidation, recent studies suggest that ferroptosis may serve as a therapeutic target for renal fibrosis [113]. SIRT1 deacetylates histones or non-histone proteins and participates in various biological processes. Upon oxalate stimulation, human kidney TECs (HK-2 cells) exhibited increased acetylation of p53. Elevated p53 acetylation promotes ferritin deposition and exacerbates renal fibrosis. SIRT1-induced deacetylation of p53 deacetylation alleviates this process. SIRT1 is a member of the HDAC3 family. However, some studies have demonstrated that protecting GPX4 by inhibiting HDAC3 can effectively reduce renal ferroptosis and delay the progression of AKI-CKD [114]. Targeting SIRT1-mediated ferroptosis is a promising research direction for the treatment of renal fibrosis (Figure 3).

## 3. Targeting SIRT1 and Its Potential Application in the Treatment of Renal Fibrosis

Resveratrol, a stilbene molecule and a member of the polyphenol family, is typically derived from diverse plant sources and is known for its antioxidant properties. It has a wide array of pharmacological effects, including antioxidant, antiaging, anti-inflammatory, antifibrotic, antidiabetic, and renoprotective activities [115,116,117]. Resveratrol can act as an activator of SIRT1. Resveratrol attenuates Con A-induced advanced glomerulosclerosis in elderly mice by upregulating Klotho expression via SIRT1 to mitigate renal oxidative stress [118]. Additionally, resveratrol ameliorates renal fibrosis by upregulating SIRT1 expression, which subsequently modulates pathways such as TGF-β/Smad3, STAT3, p53, and YY1. However, resveratrol exhibits inhibitory effects on class I, II, and IV HDACs, leading researchers to question whether the antifibrotic effects of resveratrol can be ascribed to nonspecific actions. Additional experiments are required to elucidate the role of SIRT1 activation in the regulation of renal fibrosis using more specific pharmacological activators of SIRT1.

MicroRNAs (miRNAs) are a prevalent class of endogenous short non-coding RNAs that function as post-transcriptional regulators of gene expression and play significant biological roles in both physiological and pathological processes across a spectrum of diseases. Over the past two decades, the involvement of miRNAs in renal fibrosis has garnered substantial attention, and emerging therapeutic approaches targeting miRNAs have shown considerable promise [119]. Certain miRNAs influence the development of renal fibrosis through a SIRT1-dependent mechanism. Studies have shown that MiR-34a-5p exacerbates fibrosis by directly inhibiting SIRT1, inhibition of MiR-34a-5p modulates the expression of downstream genes via SIRT1 in HK-2 cells [120]. Similarly, knockdown of miR-135a-5p attenuates TGFβ1-induced renal fibrosis by targeting SIRT1 and suppressing SMAD3 signaling, highlighting miR-135a-5p as a promising therapeutic target for diabetic nephropathy [121].

Extensive evidence suggests that natural products from traditional Chinese medicine (TCM) have significant potential in the treatment of kidney diseases [122,123]. Salidroside, an active component derived from TCM, ameliorates renal fibrosis in diabetic nephropathy by enhancing mitochondrial biogenesis through upregulation of SIRT1 and PGC-1α expression [124]. Catalpol (CAT), an iridoid glucoside derived from the roots of Rehmannia glutinosa, has been extensively used in traditional Chinese medicine for the management of diabetes, a practice that has persisted for over a millennium. Zaaba et al. [125] found that Catalpol alleviates oxidative stress and inflammation in experimental CKD through mechanisms involving SIRT1 activation and NF-κB Inhibition. Furthermore, salidroside—another TCM—activation of the SIRT1/PGC-1α pathway mitigates renal fibrosis in mice. Numerous TCMs, such as cordyceps cicadae, isoliquiritigenin, paeonol, salvianolic acid B, and Jiedu Tongluo Baoshen formula, exhibit renoprotective effects and improve renal fibrosis via SIRT1-dependent mechanisms.

Exercise is a crucial strategy for the amelioration of renal interstitial fibrosis [126,127]. Studies have demonstrated that treadmill exercise upregulates the expression of SIRT1 in the kidneys of T2DM patients. An eight-week-long treadmill exercise regimen has been shown to improve renal interstitial fibrosis in T2DM by inhibiting the TGF-β1/Smad3 pathway and reducing the expression of COL1 and COL3 proteins [128]. In Yang’s research, several significant findings have emerged: exercise training upregulates the expression of CBS/CSE in renal tissue, enhances the production of endogenous H2S, enhances the SIRT1/p53 apoptosis pathway modulation, and ameliorates diabetic nephropathy [129].

CR confers a multitude of health-promoting effects, including the extension of lifespan. One mechanism underlying the positive impact of CR is its ability to mitigate mitochondrial dysfunction in diverse pathological conditions. CR augments autophagy through the upregulation of SIRT1 in aged mammalian tissues, thereby serving as a potential therapeutic strategy for tissue damage associated with aging and hypoxia, including that observed in senescent kidneys. Fang et al. [130] revealed that SIRT1 participates in the protective effect of CR on contrast-induced nephropathy by upregulating the ferroptosis regulator glutathione peroxidase 4 (GPX4) Indeed, there is a close association between sirtuins and the myriad effects of calorie restriction. Studies have shown that extended calorie restriction for one to two years in mice augments SIRT1 expression in aging kidneys, mitigating hypoxia-induced renal injury via SIRT1-mediated deacetylation of FOXO3a and the subsequent activation of autophagy. Evidence also suggests that Reduced SIRT1 protein expression leads to increased levels of acetylated NF-κB p65, which induces renal inflammation. Conversely, dietary restriction exerted anti-inflammatory effects by restoring SIRT1 expression in the kidneys of diabetic rats.

## 4. Summary

**Figure 4:**
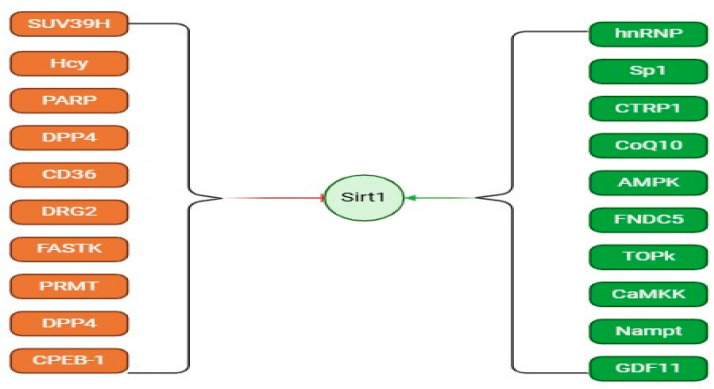
The proteins studied that can potentially activate or inhibit SIRT1. Activation and inhibition effects are displayed in green and red arrows.

Emerging data indicate that upregulation of SIRT1 expression may mitigate renal fibrosis. Collectively, SIRT1 exhibits beneficial effects in the management of renal fibrosis, suggesting its potential as a therapeutic target for this condition [131]. However, the effects of SIRT1 on different cells vary with distinct mechanisms of action. Here, we have counted some potential activations and inhibitions of SIRT1 (Figure 4).

SRT1720, a potent activator of SIRT1, promotes renal fibrosis via increased phosphorylation of epidermal growth factor receptor (EGFR) and platelet-derived growth factor receptor β (PDGFRβ). Further studies have revealed that the activation of SIRT1 promotes the activation of renal fibroblasts and exacerbates renal fibrosis. SIRT1 overexpression confers certain cells with anti-apoptotic characteristics, and its role in organ fibrosis has become a focal point of interest. Meanwhile, SIRT1 can counteract oxidative stress, inflammation, and aging by modulating pathways such as TGF-β/mTOR, FOXO, NF-κB, STAT, and the circadian clock, thereby reducing the occurrence of fibrosis.

Resveratrol, a well-known activator of SIRT1, possesses a variety of pharmacological activities, including antioxidant, anti-aging, anti-inflammatory, anticancer, antidiabetic, cardioprotective, and neuroprotective properties [132,133]. However, the application of resveratrol remains a significant challenge in the pharmaceutical industry owing to its poor solubility, low bioavailability, and adverse effects [134] The dosages used in these experiments were hundreds of times higher than those found in ordinary foods, which are unattainable for the average person. It is unrealistic to expect oral resveratrol to enhance SIRT1 activity and reduce renal fibrosis. Resveratrol inhibits all 11 human HDAC classes I, II, and IV in a dose-dependent manner, resulting in dose-dependent efficacy; however, the mechanism underlying its impact on organ fibrosis remains elusive. Future studies should focus on developing more specific SIRT1-targeted therapies and drug delivery systems for the treatment of renal fibrosis. Selective modulation of SIRT1 activation in different cell types may yield enhanced therapeutic outcomes, particularly in organs such as the kidneys, which are composed of diverse cell populations. Based on the protective effect of SIRT1 against renal fibrosis, it is important to investigate SIRT1 agonists with higher specificity.

## Figures and Tables

**Table 1:** Factors implicated in renal fibrosis.

Pathways/Factors	Action	Evidence
TGF-β1/SMAD signaling	Regulates MMT/FMT, CD32b-ERK/p38 MAP kinase crosstalk pathway; cell cycle control	[4,5,6,7]
Cell death pathways	Regulates death of tubular epithelial cells, inflammation, necroptosis, HIF-1α	[8,9,10,11]
Hypoxia pathways	Regulates mitochondrial autophagy, renal inflammation, and tubular epithelial cell apoptosis; AMPK and OPA1; EMT	[12,13,14,15]
ANG II	Regulates intracellular Ca^2+^ influx and TGF-β/SMAD2/SMAD3 signaling	[16,17,18]
Wnt/β-catenin	Regulates α-SMA expression, apoptosis, and FMT	[19,20,21]
Immunological pathways	Regulates proinflammatory cytokines and chemokines; apoptosis	[22,23]
endothelin-1	Regulates senescence in myoblasts; EMT; NF-κB	[24,25]
platelet-derived growth factor	Regulates pEMT; inflammatory mediators	[26,27]
Epidermal growth factor	Regulates CCN2 fibrotic signaling and EMT, pericyte/fibroblast migration and proliferation	[28,29]
periostin	Regulates pancreatic β-cell function; kidneyEMT; TGF-β pathway; NF-κB; PPARα.	[30,31,32,33]
galectin-3	Regulates macrophage plasticity and inflammatory process	[34,35]
Discoidindomain receptor 1	Regulates phosphorylation of BCR and STAT3; collagen-induced macrophage activation; regulates collagen transcription	[36,37]
Hedgehog Pathway	Regulates fibroblast activation and EMT; interactions exist with other signaling pathways such as TGF-β, Wnt, and Notch, which together regulate renal fiber	[38,39,40,41]

**Table 2:** The SIRT1–7’s locations and functions.

Name	Location	Function
SIRT1	nucleus cytoplasm	SIRT1 plays a role in oxidative stress-related diseases by deacetylating different target genes and proteins
SIRT2	cytoplasm	Phosphorylation during mitosis can stabilize SIRT2 and migrate into the nucleus to co-localize with chromatin. SIRT2 can serve as a checkpoint protein during cell division, inhibiting chromatin condensation and regulating cell division through H4–K16 deacetylation
SIRT3	mitochondria	Deacetylation and activation of mitochondrial matrix enzymes regulate the transcription of gluconeogenesis-related genes
SIRT4	mitochondria	Plays a role in energy metabolism and coping with stress responses
SIRT5	mitochondria	Plays a role in energy metabolism and coping with stress responses
SIRT6	nucleus	Participate in DNA repair and cellular stress response
SIRT7	nucleus	The function is still under research
